# A Bibliometric Analysis of 34,692 Publications on Thyroid Cancer by Machine Learning: How Much Has Been Done in the Past Three Decades?

**DOI:** 10.3389/fonc.2021.673733

**Published:** 2021-10-14

**Authors:** Zeyu Zhang, Lei Yao, Wenlong Wang, Bo Jiang, Fada Xia, Xinying Li

**Affiliations:** Department of Thyroid Surgery, Xiangya Hospital, Central South University, Changsha, China

**Keywords:** thyroid cancer, bibliometrics, machine learning, natural language processing, latent Dirichlet allocation

## Abstract

**Introduction:**

Thyroid cancer (TC) is the most common neck malignancy. However, a large number of publications of TC have not been well summarized and discussed with more comprehensive methods. The purpose of this bibliometric study is to summarize scientific publications during the past three decades in the field of TC using a machine learning method.

**Material and Methods:**

Scientific publications focusing on TC from 1990 to 2020 were searched in PubMed using the MeSH term “thyroid neoplasms”. Full associated data were downloaded in the format of PubMed, and extracted in the R platform. Latent Dirichlet allocation (LDA) was adopted to identify the research topics from the abstract of each publication using Python.

**Results:**

A total of 34,692 publications related to TC from the last three decades were found and included in this study with an average of 1,119.1 publications per year. Clinical studies and experimental studies shared the most proportion of publications, while the proportion of clinical trials remained at a relatively small level (5.87% as the highest in 2004). Thyroidectomy was the lead MeSH term, followed by prognosis, differential diagnosis, and fine-needle biopsy. The LDA analyses showed the study topics were divided into four clusters, including treatment management, basic research, diagnosis research, epidemiology, and cancer risk. However, a relatively weak connection was shown between treatment managements and basic researches. Top 10 most cited publications in recent years particularly highlighted the applications of active surveillance in TC.

**Conclusion:**

Thyroidectomy, differential diagnosis, genomic analysis, active surveillance are the most concerning topics in TC researches. Although the BRAF-targeted therapy is under development with promising results, there is still an urgent need for conversions from basic studies to clinical practice.

## Introduction

Thyroid cancer (TC) is the most common neck malignancy and is on the rise to become the fourth leading type of cancer worldwide (1). During the past decades, great progress and significant developments have been achieved in the field of TC with a growing number of publications. These scientific publications can reflect the hotspots of researches and suggest the future research direction (2). A bibliometric analysis is often used to quantify the progress of the field through published literature. The most recent bibliometric analyses by Cooper et al. summarized 19,055 articles from 2006 to 2015 in the broad area of clinical thyroid disease (3). However, the large amount of publication data in the special field of TC have not been well summarized and discussed with more comprehensive methods.

Natural language processing (NLP) is a series of machine learning methods for human language analyses. Among these methods, the Latent Dirichlet allocation (LDA) is most usually used to analyze scientific publications by identifying specific themes and dividing documents into these themes (4–6). In addition, LDA has been used in bibliometric analyses in the field of rectal cancer and gliomas (7, 8).

The present study is aiming at summarizing scientific publications of TC in the past three decades. Moreover, by detailedly analyzing the research topics using a machine learning method, we are hoping to provide insights into scientific developments, recognize areas with declining interest, and more importantly, reveal potential future research foci in the field of TC.

## Materials and Methods

Scientific publications focusing on TC from 1990 to 2020 were searched in PubMed using the MeSH term “thyroid neoplasms” on January 26, 2021, limited by the language of English. Full associated data were downloaded in the format of PubMed, and extracted in the R platform using the package “Bibliometrix”, (9) including the publication year, the publication type, MeSH terms, abstract, etc. MeSH terms appearing more than 25 times were included in analyses. In addition, the present study did not need ethical approval from an institutional review board or ethics committee because this was a bibliometric analysis.

LDA was adopted to identify the research topics from the abstract of each publication using Python. A feature glossary of terms was created by LDA according to the frequency of coexistence of vocabulary words in the publication series. Subsequently, LDA would calculate the most two probable research topics of each publication, depending on the frequency of appearance of these glossary words in each publication. In order to further investigate the relationship between these topics, a cluster analysis was performed using the Louvain algorithm.

The visualizations were achieved by R platform and Excel, while the network was constructed by Gephi (https://gephi.org/) (10). All the mentioned codes, including R code and Python code, were available on GitHub (https://github.com/yan-wen0614/Medicine-Bibliometric-Analysis).

## Results

**Figure 1:**
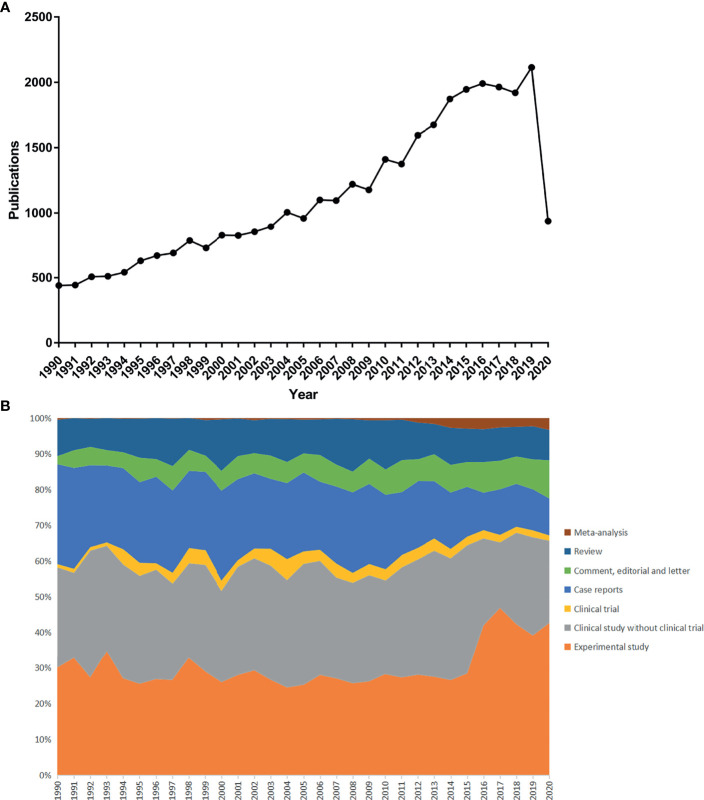
**(A)** Scientific publications per year; **(B)** Distribution of publication types per year.

**Figure 2:**
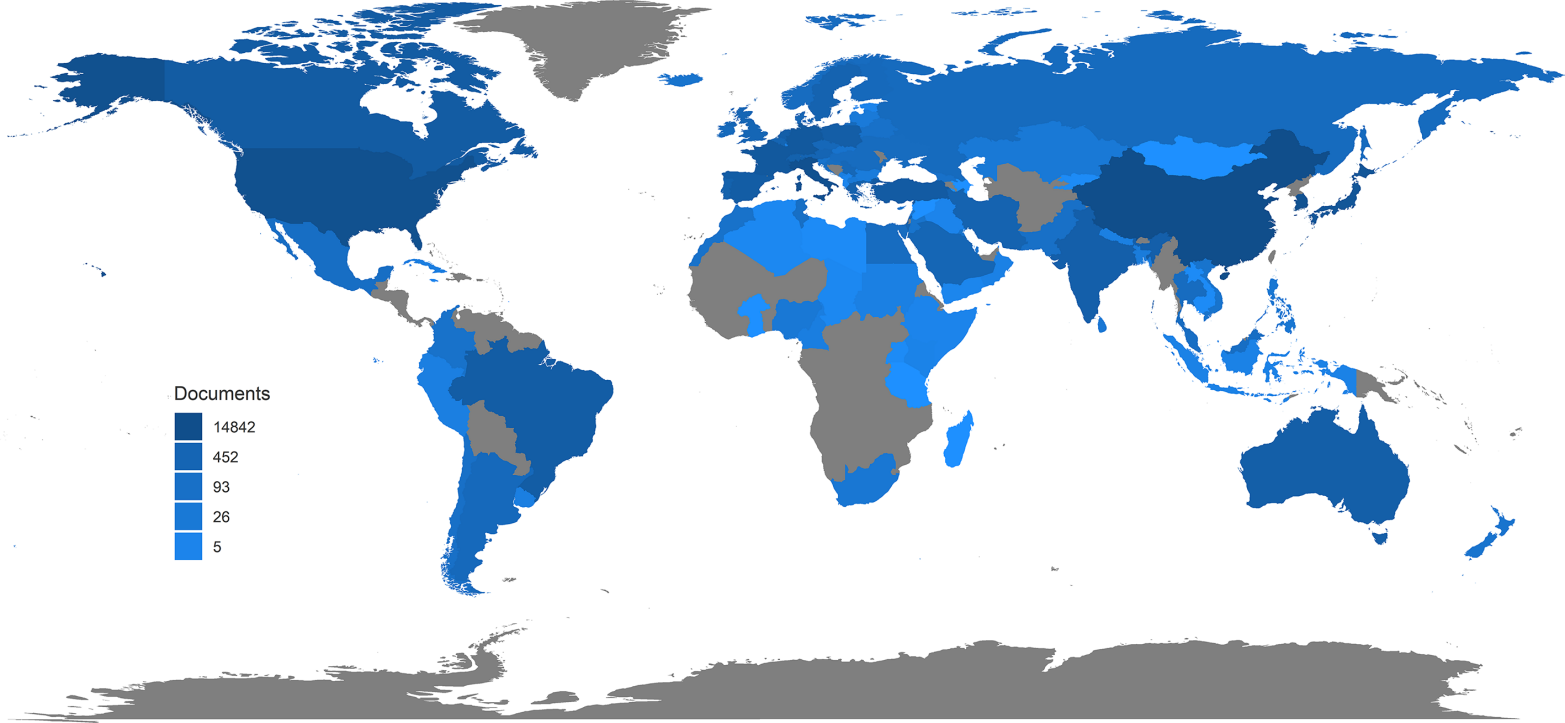
Country-scientific production.

**Figure 3:**
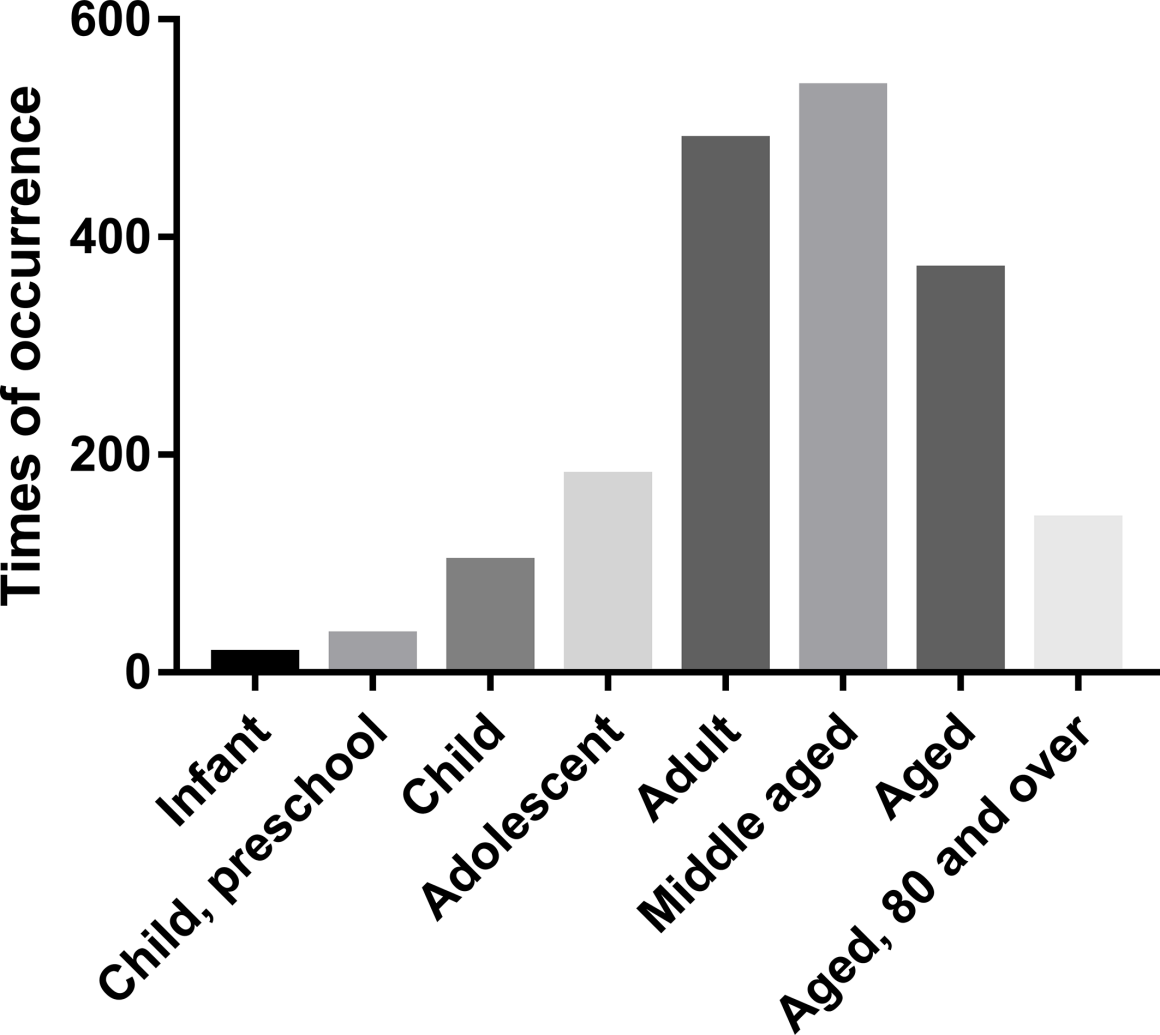
The mean times of occurrence of MeSH terms concerning age per year.

A total of 34,692 publications related to TC from the last three decades were found and included in this study with an average of 1,119.1 publications per year. As shown in **Figure 1A**, the quantity of TC publications continuously raised during the past three decades and was plateauing since 2014. However, it declined to 937 in 2020, where the pandemic of COVID-19 may take the responsibility. The distribution of publication types is shown in **Figure 1B**. Clinical studies and experimental studies shared the most proportion of publications. With the development of evidence-based medicine, the proportion of meta-analysis gradually increased during these years, while the proportion of clinical trials remained at a relatively small level (5.87% as the highest in 2004). Moreover, the country-scientific production is shown in **Figure 3**. China, USA, Italy, South Korea, and Japan were the top five countries producing TC-related publications (**Figure 2**). In addition, the top 10 affiliations with the highest scientific production are listed in **Table 1**.

**Table 1:** Top 10 affiliations with highest scientific production.

Name of affiliation	Number of articles
University Of Ulsan College of Medicine	1,032
Yonsei University College of Medicine	964
University of Pisa	876
Sungkyunkwan University School of Medicine	739
Memorial Sloan Kettering Cancer Center	664
National Cancer Institute	657
Harvard Medical School	638
Fudan University	630
Kuma Hospital	564
Tongji University School of Medicine	508

### MeSH Term Analyses

A total of 1,595 MeSH terms with 3,459,152 times of occurrence were included in detailed MeSH term analyses.

MeSH terms concerning the age of the study population were initially investigated (**Figure 3**). Eight age groups were identified as follows: infant (birth to 2–3 months), preschool child (2–5 years), child (6–12 years), adolescent (13–18 years), adult (19–44 years), middle-aged (45–64 years), aged (65–79 years), and aged (80 years and over). Overall, the middle-aged was the most predominant cohort in TC studies, as 536.94 occurrence times per year.

**Figure 4:**
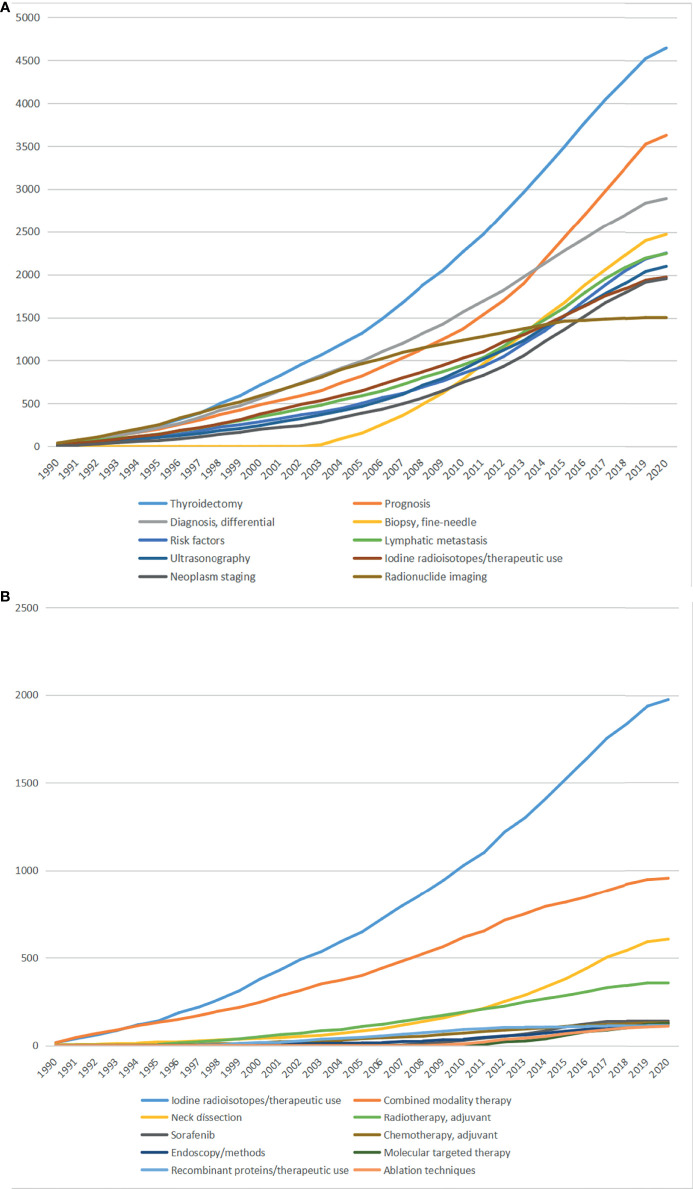
**(A)** Top 10 MeSH terms and accumulative occurrences during the past three decades; **(B)** Top 10 MeSH terms concerning treatments of thyroid cancer and accumulative occurrences during the past three decades.

**Figure 4A** shows the top 10 MeSH terms, representing the most concerned research foci. Thyroidectomy was the lead term, followed by prognosis, differential diagnosis, fine-needle biopsy, etc. In 2014, the researches focusing on prognosis surpassed the researches focusing on differential diagnosis, suggesting the focus of TC researches might move to treatment outcomes from the diagnosis of TC. Notably, fine-needle biopsy firstly appeared in 2000, however rapidly growing as the third hot field in 2020.

In order to acknowledge the role of TC treatments, we further identified the top 10 MeSH terms associated with TC treatments except for thyroidectomy (**Figure 4B**). Iodine radioisotopes was the lead term of TC treatments, followed by combined modality therapy, neck dissection, adjuvant radiotherapy, etc. Among them, molecular targeted therapy, including sorafenib, and ablation therapy appeared after 2003. Additionally, iodine radioisotopes and neck dissection were the fastest growing fields.

### LDA Analyses

**Figure 5:**
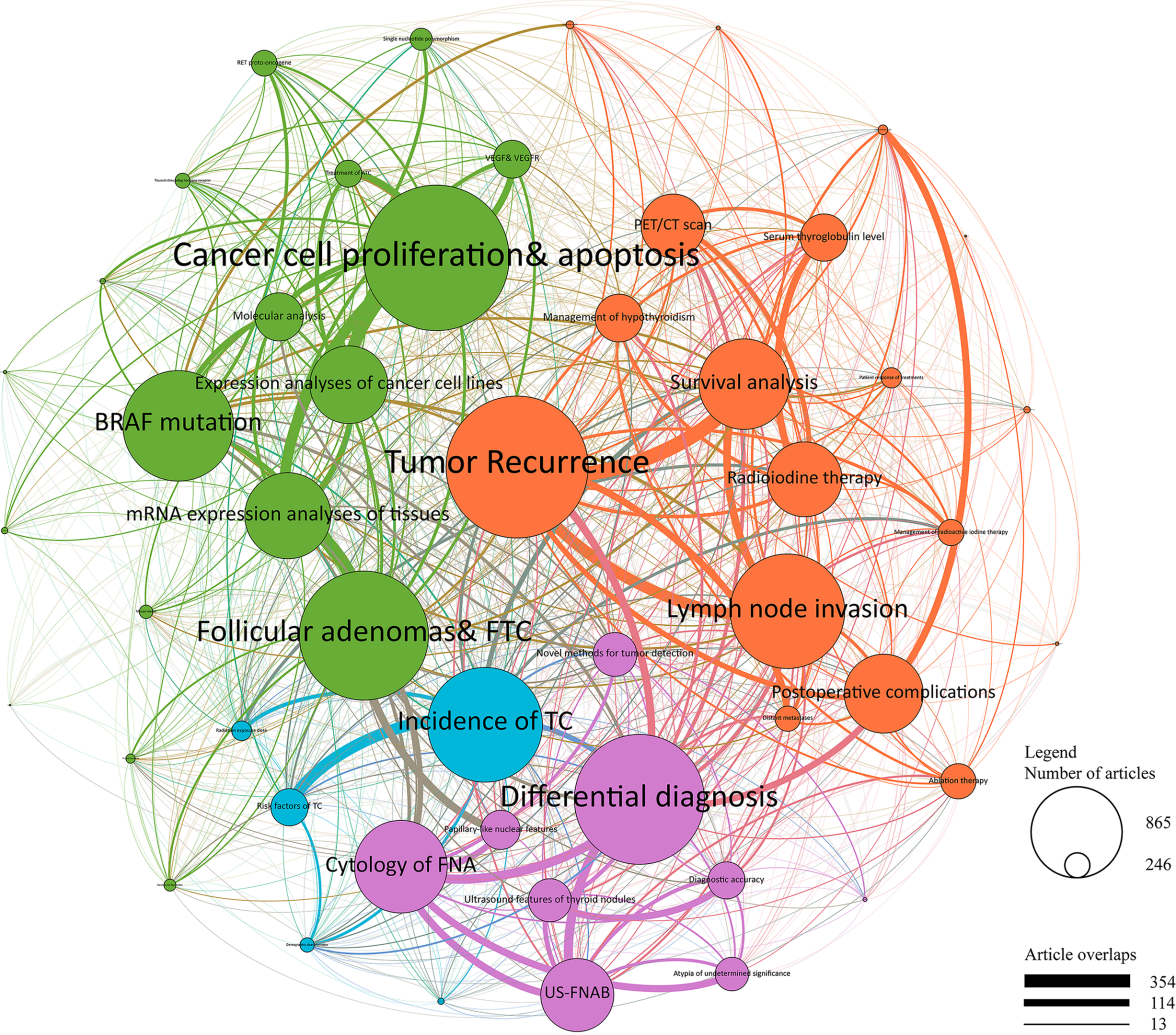
Topic cluster network by Latent Dirichlet Allocation. Red, Treatment management; Green, Basic research; Purple, Diagnosis research; Blue, Epidemiology and cancer risk. The size of circle represents the number of papers in each topic, and the thickness of line represents the weight of connection between each topic.

A total of 21,491 publications were further analyzed using the LDA method, excluding publications without an abstract. Using the text of the abstract, LDA analyses provided further information about hot topics and their relationships by constructing a topic network (**Figure 5**). The network was divided into four clusters, including treatment management (in red), basic research (in green), diagnosis research (in purple), and epidemiology and cancer risk (in blue). The focalization of topics and the weight of connections between topics were also shown as the size of the circle and the thickness of the line, respectively.

In the cluster of treatment management, tumor recurrence, lymph node invasion, and survival analysis were the top three studied topics. Great efforts were also made in the field of postoperative complications, radioiodine therapy, positron emission tomography/computed tomography (PET/CT) scan, and ablation therapy. In the cluster of diagnosis research, differential diagnosis, cytology of fine-needle aspiration (FNA), and ultrasound-guided fine needle aspiration biopsy (US-FNAB) were the top three studied topics. Strong connections were shown between these two clusters, especially between differential diagnosis and tumor recurrence. The cluster of epidemiology and cancer risk covered a relatively small proportion of topics, however having high connectivity with the cluster of treatment management and the cluster of diagnosis research. Moreover, cancer cell proliferation and apoptosis, BRAF mutation, expression analyses of cell lines and tissues accounted for the main part of the basic research cluster. Notably, the topic of follicular adenomas and follicular thyroid carcinoma (FTC) also had a great significance in the basic research cluster, which was strongly connected with the cluster of diagnosis research. However, the rest of the basic research cluster showed a relatively poor connection with other clusters, suggesting that there may be an urgent need for conversions from basic researches to clinical treatments.

### Top-Cited Publications

Additional attention was put on recent top-cited publications. The top 10 most cited original researches from 2016 to 2020 were searched, while review, guidelines, and epidemiological papers were excluded. The results are listed in **Table 2**. Among these papers, three (1st, 5th, 7th) were concerning the overtreatment and active surveillance; two (2nd, 8th) were concerning the genomic and transcriptomic analyses; three (3rd, 6th, 9th) were concerning the surgical procedures; one (4th) was concerning the differential diagnosis, while one (10th) was concerning the targeted therapy of advanced TC.

**Table 2:** Top 10 most cited publications in the field of thyroid cancer.

Title	Journal	Citations
Nomenclature Revision for Encapsulated Follicular Variant of Papillary Thyroid Carcinoma: A Paradigm Shift to Reduce Overtreatment of Indolent Tumors	JAMA ONCOLOGY	723
Genomic and transcriptomic hallmarks of poorly differentiated and anaplastic thyroid cancers	JOURNAL OF CLINICAL INVESTIGATION	459
Transoral Endoscopic Thyroidectomy Vestibular Approach: A Series of the First 60 Human Cases	WORLD JOURNAL OF SURGERY	213
Impact of reclassifying noninvasive follicular variant of papillary thyroid carcinoma on the risk of malignancy in The Bethesda System for Reporting Thyroid Cytopathology	CANCER CYTOPATHOLOGY	177
Natural History and Tumor Volume Kinetics of Papillary Thyroid Cancers During Active Surveillance	JAMA OTOLARYNGOLOGY-HEAD & NECK SURGERY	174
Is There a Minimum Number of Thyroidectomies a Surgeon Should Perform to Optimize Patient Outcomes?	ANNALS OF SURGERY	168
Incidences of Unfavorable Events in the Management of Low-Risk Papillary Microcarcinoma of the Thyroid by Active Surveillance *Versus* Immediate Surgery	THYROID	168
Genetic Analysis of 779 Advanced Differentiated and Anaplastic Thyroid Cancers	CLINICAL CANCER RESEARCH	149
Safety and Outcomes of the Transoral Endoscopic Thyroidectomy Vestibular Approach	JAMA SURGERY	148
Vemurafenib in patients with BRAF(V600E)-positive metastatic or unresectable papillary thyroid cancer refractory to radioactive iodine: A non-randomised, multicentre, open-label, phase 2 trial	LANCET ONCOLOGY	137

## Discussion

For the very first time, the present study comprehensively investigated 34,692 scientific publications concerning TC during the past three decades using a machine learning method. The number of publications in TC expanded rapidly, however with a small proportion of clinical trials. The most concerning topics of TC researches were thyroidectomy, differential diagnosis, genomic analysis, active surveillance. Furthermore, the results of LDA analyses also indicated a potential need for conversions of novel therapeutic targets from basic researches to treatment management. The top 10 most cited papers also covered these hot topics.

There is no doubt about the predominant role of thyroidectomy in the field of TC treatment. With the increasing incidence of TC, thyroidectomy is subsequently increasing (11). Recurrence is a critical issue when it comes to patient prognosis after thyroid surgery. A Modified Initial Risk Stratification system was proposed in the 2015 ATA guidelines, depicting the risk of recurrence in differentiated thyroid carcinoma (DTC) patients after initial therapy (12). Furthermore, multiple treatments are also taken to inhibit tumor recurrence, including thyroid stimulating hormone (TSH) suppression therapy and radioactive iodine (RAI) therapy (13). Another hot topic in TC treatment is lymph node invasion, as the lymph node metastases are reported in up to 50% of patients with TC (14). Lymph node invasion is proven to be associated with a higher recurrence rate and poorer survival data (15). While the lymphadenectomy is necessary for patients detected with suspicious lymph node invasion, the role of prophylactic central compartment neck dissection (pCCND) still remains controversial, mainly because of the unclear long-term outcomes of pCCND (16). In addition, novel surgical approaches, including intraoperative nerve monitoring, transoral and robotic approaches, are developing in these recent years (17, 18). Although unfortunately, they were not detected in MeSH term analyses or LDA analyses because of novelty, we still value their development prospects in further thyroidology.

Differential diagnosis and US-FNAB were the main topics in the diagnosis research cluster. In fact, differential diagnosis always plays as the central issue in diagnosing TC, with US-FNAB as an extremely important method. US-FNAB is simple, safe, and reliable, providing the most definitive information for differential diagnosis. In most of the medical centers in the world, cytology results from US-FNAB are reporting using six categories of the 2017 updated Bethesda classification system (19). However, there are still 20 to 30% of patients with indeterminate results, requiring additional evaluations (20). As a novel diagnostic method, genomic analysis, including micro-RNA analysis, holds a great potential in contributing to the differential diagnosis (21). Future diagnosing system should take the genomic analysis into considerations. Meanwhile, the basic research cluster also showed some associations between BRAF mutation and the treatment management cluster, suggesting the potential of BRAF-targeted therapies in treating TC. However, other parts of basic research showed scarce connections with the treatment management cluster, indicating an urgent need for more conversions from basic studies to clinical treatments.

This study highlighted topics of thyroidectomy, differential diagnosis, genomic analysis, translational researches in the field of TC. Meanwhile, the top 10 most cited papers seemed to concern consistent fields. Moreover, the overtreatment issue and the active surveillance were particularly raised by most cited papers. These studies showed a large proportion of papillary thyroid cancer did not need surgical intervention. However, the problems mainly lie in effective methods to sort out the patients who need immediate surgery, and a unified standard to suggest surgery to patients who suffer from progressed lesions. The overtreatment issue may be simultaneously settled when the problems are solved. Additionally, the results of the present study showed a lack of high-quality RCT, and there was only one non-randomized study in the top 10 most cited papers. Although TC holds a relatively higher incidence than most other cancer types, most TC patients do not need any treatments other than surgery. The rest minority with advanced diseases can be further divided into several subtypes with potentially different therapeutic needs due to various pathogenic mechanisms, such as poorly differentiated TC, anaplastic TC, radioactive iodine refractory TC, etc., causing the difficulties of therapy developments, as well as a relatively small number of trial candidates in each subtype. It may be the reason for lacking high-quality researches.

There were also some limitations in this study. Firstly, only PubMed was chosen for this bibliometric research because of the high quality and comprehensive information of publications, while other databases were also available. Secondly, the MeSH term was used for searching publications, which might restrict the comprehensiveness of our search. Lastly, some publications were tagged with multiple publication types. Although the publication type was manually adjusted with double-check, it might still cause inaccuracy.

## Conclusion

Thyroidectomy, differential diagnosis, genomic analysis, active surveillance are the most concerning topics in TC researches. Although the BRAF-targeted therapy is under development with promising results, there is still an urgent need for conversions from basic studies to clinical practice.

## Data Availability Statement

The original contributions presented in the study are included in the article/supplementary materials. Further inquiries can be directed to the corresponding author.

## Author Contributions

All authors made substantive intellectual contributions to this study to qualify as authors. XL and FX conceived the design of the study. FX modified the design of the study. ZZ, FX, WW, and LY performed the study, collected the data, and contributed to the design of the study. ZZ, LY and BJ analyzed the data. ZZ and LY drafted the *Result*, *Discussion*, and *Conclusion* sections. FX and WW drafted the *Methods* sections. FX, XL, and LY edited the manuscript. All authors read and approved the final manuscript. All authors have agreed to be accountable for all aspects of the work in ensuring that questions related to the accuracy or integrity of any part of the work are appropriately investigated and resolved.

## Funding

This work was supported by the National Natural Science Foundation of China (grant No. 82073262) and the Hunan Province Natural Science Foundation (grant number 2019JJ40475).

## Conflict of Interest

The authors declare that the research was conducted in the absence of any commercial or financial relationships that could be construed as a potential conflict of interest.

## Publisher’s Note

All claims expressed in this article are solely those of the authors and do not necessarily represent those of their affiliated organizations, or those of the publisher, the editors and the reviewers. Any product that may be evaluated in this article, or claim that may be made by its manufacturer, is not guaranteed or endorsed by the publisher.
